# Ghrelin protects against contact dermatitis and psoriasiform skin inflammation by antagonizing TNF-α/NF-κB signaling pathways

**DOI:** 10.1038/s41598-018-38174-2

**Published:** 2019-02-04

**Authors:** Ruize Qu, Xiaomin Chen, Jing Hu, Yufeng Fu, Jiangfan Peng, Yuhua Li, Jingxi Chen, Peng Li, Long Liu, Jiankang Cao, Wenhan Wang, Cheng Qiu, Linlin Guo, Krasimir Vasilev, Jianying Chen, Gengyin Zhou, Weiwei Li, Yunpeng Zhao

**Affiliations:** 1 Department of Pathology, Qilu Hospital, Shandong University, Jinan, China; 2 Cheeloo College of Medicine, Shandong University, Jinan, China; 3 Department of Obstetrics and Gynecology, Qilu Hospital, Shandong University, Jinan, China; 4 Department of Orthopedics, Qilu Hospital, Shandong University, Jinan, China; 5 School of Engineering, University of South Australia, Mawson Lakes, South Australia Australia; 6 Institute of Biopharmaceuticals of Shandong Province, Jinan, China

## Abstract

Contact dermatitis and psoriasis are skin disorders caused by immune dysregulation, yet much remains unknown about their underlying mechanisms. Ghrelin, a recently discovered novel peptide and potential endogenous anti-inflammatory factor expressed in the epidermis, is involved in skin repair and disease. In this study, we investigated the expression pattern and therapeutic effect of ghrelin in both contact dermatitis and psoriasis mouse models induced by oxazolone (OXA) and imiquimod (IMQ), respectively, and in TNF-α-stimulated RAW264.7 macrophages, NHEKs and skin fibroblasts. Ghrelin expression was reduced in both the OXA-induced contact dermatitis and IMQ-induced psoriasis mouse models. Furthermore, treatment with ghrelin attenuated skin inflammation in both the contact dermatitis and psoriasis mouse models. Mice administered PBS after OXA- or IMQ-induced model generation exhibited typical skin inflammation, whereas ghrelin treatment in these mouse models substantially decreased the dermatitis phenotype. In addition, exogenous ghrelin attenuated the inflammatory reaction induced by TNF-α in RAW264.7 cells. Moreover, ghrelin administration limited activation of NF-κB signaling. In summary, ghrelin may represent a potential molecular target for the prevention and treatment of inflammatory skin diseases, including contact dermatitis and psoriasis.

## Introduction

Dermatitis, including contact dermatitis and psoriasis, is a common inflammatory skin disorder affecting millions of people worldwide^1,2^. As the largest organ in the body, the skin is the first natural barrier of the immune system, and inflammatory diseases of the skin can lead to easier infection by bacteria and viruses. Currently, the precise mechanisms underlying the pathogenesis and effective therapies for contact dermatitis and psoriasis remain unknown^3–5^. Contact dermatitis is characterized by erythema, swelling, papules, blister and bullaeand, and psoriasis is characterized by hyperproliferation of keratinocytes, enlargement and growth of dermal capillary vasculature, and infiltration of T lymphocytes and neutrophils into the dermis and epidermis. Additionally, cytokine and chemokine levels are increased in the skin and circulation of both contact dermatitis and psoriasis patients^6,7^. Contact dermatitis and psoriasis models induced by oxazolone (OXA) and imiquimod (IMQ), respectively, harboring significant phenotypic and histological similarities to human contact dermatitis and psoriasis, have been widely used to evaluate potential therapeutics in these diseases^8–10^.

TNF-α is a well-known inflammatory factor involved in a variety of inflammatory diseases^11–13^. It is primarily produced by macrophages during inflammation and exerts negative effects^14–16^. It has been reported that TNF-α mainly takes part in inflammation through autocrine and paracrine activation of macrophages to increase the generation of inflammatory cytokines, including IL-1β, IL-6, COX-2 and iNOS, which can lead to a chain reaction of inflammation^17–19^. Moreover, TNF-α has been reported to be part of the inflammatory process of skin inflammatory diseases, and inhibition of TNF-α yields positive effects on the treatment of dermatitis^20–23^.

Ghrelin is a novel peptide mainly produced by X/A-like cells of the stomach but is also detected in numerous additional tissues^24^. Ghrelin acts as a critical factor in a variety of physiological and disease processes, including neurogenesis, tumorigenesis, hypertension and tissue regeneration^25–29^. Additionally, the anti-inflammatory function of ghrelin has been extensively studied^30–35^. Its mechanism might be mediated through inhibition of inflammatory cytokines by binding to its receptor, GHSR1a, thus protecting the body from inflammation^35–37^. Notably, ghrelin has been found to protect against TNF-α-induced inflammatory conditions^38^. Given the critical role of TNF-α in the initiation and progression of inflammation and the positive role reported for ghrelin in some inflammatory diseases, in this study, we aimed to examine whether ghrelin affects contact dermatitis and psoriasis by limiting TNF-α and to explore its molecular mechanisms, which may highlight a new direction of study for the treatment of both contact dermatitis and psoriasis.

## Materials and Methods

### Animals

Three-month-old C57BL/6 mice were purchased from Shandong University. The animals were treated for 7 days in a standard environment (23 ± 2 °C, 12-hour light/dark cycle). All experiments were carried out in accordance with institutional guidelines and were approved by the Institutional Animal Care and Use Committee of Shandong University.

### Cell culture

RAW264.7 cells are an immortalized murine macrophage cell line, and normal human epidermal keratinocytes (NHEKs) are a human epidermal cell line. In this study, we utilized RAW264.7, NHEK cells as well as mice skin fibroblast cells, which were purchased from the Type Culture Collection of the Chinese Academy of Sciences, Shanghai, China, for *in vitro* experiments. Cells were cultured at 37 °C in 5% CO2 in Dulbecco’s modified Eagle’s medium (Gibco, U.S.A.) with 10% fetal bovine serum (Gibco, U.S.A.) and 1% penicillin–streptomycin (HyClone, UT, U.S.A.). After being cultured for 2–3 days, the cells were replated at 80% confluence in 6-well plates before use for further analysis. For experiments, cells were stimulated with TNF-α (10 μg/ml, R&D Systems, NE, U.S.A.) in the absence or presence of varying levels of ghrelin (sc-364689, Santa Cruz Biotechnology, U.S.A.). After 1 day of incubation, cells and culture supernatant were collected for further analysis.

### Skin culture

Mouse skin tissues were collected from dorsal skin of newborn C57BL/6 mice. Skin was cut into thin slices (100–300 μm) with a microtome, and organs were then cultured in DMEM with 10% FCS, gentamicin (15.2 µg/ml) and ciproxin (19 µg/ml) at 37 °C in 5% CO2, as previously described^39,40^. For further experiments, organs were stimulated with TNF-α (10 μg/ml, R&D Systems) with or without ghrelin treatment (sc-364689, Santa Cruz Biotechnology, U.S.A.). After culturing for 3 days, organs were collected for the following examinations.

### Experiment 1

The contact dermatitis model was established as previously reported^7^. In short, mice were sensitized by a single application of 50 μl of 1.5% oxazolone (Sigma, MO, U.S.A.) in ethanol to dorsal skin for 7 days, following application of 20 μl of 1.5% oxazolone in ethanol on the ear lobes every day or every other day for 14 days. Ear thickness was measured with a micrometer (Mitutoyo, Kanagawa, Japan) every other day. Mice were sacrificed on the 14th day, and tissue samples were collected for further analysis.

### Experiment 2

Mice were shaved on the back, and some mice were treated locally with IMQ (5%) cream (Mingxin Pharmaceuticals, Sichuan, China) at 40 mg per day on dorsal skin for 7 days. Dorsal skin tissues and blood samples were collected afterward for further analysis.

### Grouping and treatment

C57BL/67 mice were randomly divided into 3 groups for each experiment: 1. IMQ/OXA + ghrelin group (treated with IMQ or OXA and ghrelin) (n = 7). Mice received an intraperitoneal injection of ghrelin (1 µg/g body weight, sc-364689, Santa Cruz Biotechnology, U.S.A.) dissolved in PBS every day, along with IMQ or OXA. 2. IMQ/OXA + PBS (treated with IMQ or OXA and PBS) (n = 7). IMQ/OXA-treated mice received an injection of the same volume of PBS as ghrelin in Group 1 for 7 days. 3. CTL group (treated with Vaseline/ethanol and PBS) (n = 7). Mice received an intraperitoneal injection of PBS, and Vaseline/ethanol was applied to the dorsal skin or ear lobes.

### Severity scoring of skin inflammation

To examine the level of inflammation due to psoriasis on dorsal skin in mice, an objective scoring system was designed based on the clinical psoriasis area and severity index (PASI), although the affected skin area was not considered in the overall score, as was previously reported^33^. Once daily, the degree of erythema, scaling and thickening were evaluated on dorsal skin using a five-point scale: 0, none; 1, slight; 2, moderate; 3, marked; and 4, very marked. The PASI score was calculated by adding the scores for the 3 separate criteria, thus allowing a range from 0 to 12. The results were averaged for each group, and trend lines were created to display the skin lesion changes.

### Isolation of epidermis and dermis

To examine the expression pattern of ghrelin in epidermis and dermis separately, epidermis and dermis were isolated from mice dorsal skin as was previously described^41^. In short, skin tissues of mice were cut into thin strips of about 1 mm, soak it in a sterile 0.25% dispase solution and then stay overnight at 4 °C. The dermis and epidermis were following separated with ophthalmic tweezers. The isolated tissues were stored at −80 °C for further analysis.

### Histology

Dorsal skin and mouse ear samples were collected from each experimental group, fixed in 10% formalin, dehydrated, and then cleared with dimethylbenzene. Thereafter, the samples were embedded in olefin. At least four consecutive sections were cut from sagittal planes and then stained with H&E for routine histological analysis. The thickness of the epidermis was then determined for Experiment 1 by using OSTEOMEASURE software (OsteoMetrics, Inc., Decatur, GA, U.S.A.).

### Immunohistochemistry

Tissues from each experimental group of the 2 models were collected, fixed for 48 hours in 10% formalin and then prepared and incubated with polyclonal anti-CD4, anti-CD68, anti-iNOS, anti-p-IκBα and anti-ghrelin serum (anti-CD4 antibody: 1:100 dilution, sc-70670, Santa Cruz Biotechnology, U.S.A.; anti-CD68 antibody: 1:100 dilution, sc-7084, Santa Cruz Biotechnology, U.S.A.; anti-iNOS antibody: 1:150 dilution, 18985-1-AP, Proteintech Biotechnology, U.S.A.; p-IκBα: 1:100 dilution, sc-101713, Santa Cruz, U.S.A.; anti-ghrelin antibody: 1:100 dilution, sc50297, Santa Cruz Biotechnology, U.S.A.^32^) at 4 °C overnight, followed by incubation with horseradish peroxidase-conjugated secondary antibody (Zhongshanjinqiao Biotechnology, P.R., China) for 60 minutes at RT. Signal was detected by using the Vector Elite ABC Kit (Vectastain; Vector).

### Immunofluorescence staining

Skin tissues were gathered, stained and paraffin embedded, following 5 μm thick sections were cut. Immunofluorescence staining was then performed to testify ghrelin expression with anti-ghrelin antibody (1:100 dilution, sc50297, Santa Cruz Biotechnology, U.S.A.), as described previously, and examined using a confocal fluorescence microscope system^42^.

### Western blot analysis

Total protein was isolated from cells and tissues using cell lysis buffer and then centrifuged at 12000 rpm for 6 minutes at 4 °C. Protein concentrations in the supernatants were measured using a BCA protein assay kit (Beyotime Corporation, Shanghai, China). Equal amounts of protein were separated by SDS-PAGE and transferred to PVDF membranes. After blocking with 5% BSA for 1 hour at room temperature, the membranes were incubated with primary antibodies against β-tubulin (1:1500 dilution, #2148, Cell Signaling Technology, U.S.A.), iNOS (1:1000 dilution, ab15323, Abcam Corporation, U.S.A.), ghrelin (1:500 dilution, sc-50297, Santa Cruz, U.S.A.), IκBα (1:1000 dilution, ab32518, Abcam, U.S.A.) and p-IκBα (1:1000 dilution, sc-101713, Santa Cruz, U.S.A.) at RT for 1–2 hours, followed by application of appropriate HRP-conjugated secondary antibodies for 1 hour at RT. Immunoreactive bands were imagined with a DNR Bio-Imaging system based on the manufacturer’s instructions. The expression of cytoplasmic protein was normalized to β-Actin or β-tublin using ImageJ software.

### Real-time quantitative PCR

Total mRNA was extracted from RAW264.7 cell and cells from the dorsal skin or ear lobe samples with TRIzol Reagent (Takara Biotechnology, Otsu, Japan). Following extraction, reverse transcription was completed to convert total RNA to cDNA with the PrimeScript RT Reagent Kit (Takara Biotechnology, Otsu, Japan) following the manufacturer’s protocol. PCR was performed with an RNA PCR kit (Takara Biotechnology, Otsu, Japan). The 2−ΔΔCT method was utilized to quantify the data. The PCR primer sequences are listed in Supplementary Table 1.

### Enzyme-linked immunosorbent assay (ELISA)

The concentrations of IL-1β and IL-6 in the sera of C57BL/6 mice and cell culture media from RAW264.7 cells were measured by ELISA according to the manufacturer’s instructions (ELISA kits for IL-1β: eBioscience, Frankfurt, Germany; ELISA kits for IL-6: R&D Systems, Minneapolis, MN, U.S.A.). All assays were performed in duplicate, as previously reported^29^.

### Nitrite production assay

To examine whether ghrelin inhibits inflammatory reactions mediated by TNF-α in RAW264.7 cells, a Griess assay was conducted as previously described^43,44^. In short, RAW264.7 cells were cultured in 96-well flat-bottom plates with 10 ng/ml TNF-α (R&D Systems, NE, U.S.A.) for 24 hours with or without a concentration gradient of ghrelin treatment. Cell culture supernatants were then collected and examined for NO using the Griess reaction with a commercial kit.

### Statistical analysis

The data are presented as the mean ± SD, and GraphPad Prism v. 6.0 software was used for statistical analyses. Student’s t-test or one-way analysis of variance (ANOVA) was employed to determine the statistical significance of differences. A value of P < 0.05 was regarded as statistically significant.

## Results

### Ghrelin is found in macrophages and epidermal cells and is diminished in response to inflammatory cytokines in the skin

Ghrelin is a novel peptide mainly produced by X/A-like cells of the stomach, but is also found in many additional organs and secreted by many additional cell types. Additionally, ghrelin is reportedly expressed in many cell types found in the skin, including epidermal cells, lymphocytes and macrophages. Reports have shown that the production and loss of ghrelin are closely linked to inflammatory reactions in many diseases^32,45^. In this study, we first characterized the expression pattern of ghrelin in the skin. RAW264.7 cells and NHEKs were cultured and then stimulated with TNF-α for 24 hours. Real-time PCR was conducted with mRNA extracted from RAW264.7 cells and NHEKs. As shown in Supplementary Figs 1A,D and 2A, ghrelin was found in both of these cell lines and was diminished upon stimulation with TNF-α. In addition, the total proteins for each experimental group with these three types of cells were collected, and western blot was conducted to assess ghrelin expression. As shown in Supplementary Figs 1B,C,E,F and 2B,C, ghrelin expression was detected in both RAW264.7 cells, NHEKs and mice skin fibroblast cells and was diminished upon stimulation with TNF-α. Next, a piece of dorsal skin was taken, cut into slices in DMEM in a sterile environment, and then treated with inflammatory cytokines, such as TNF-α, at 37 °C for 3 days. Tissues were collected and prepared, and immunohistochemistry was performed to examine ghrelin expression. As shown in Supplementary Fig. 1G, ghrelin secretion was evaluated in skin tissues, and stimulation with TNF-α greatly decreased ghrelin levels in the skin. Moreover, mRNA was collected from the skin tissue of each experimental group, and real-time PCR was performed. The results showed that ghrelin expression was dramatically decreased upon TNF-α stimulation (Supplementary Fig. 1H). Total protein was subsequently extracted from cultured tissues, and western blotting was conducted. As shown in Supplementary Fig. 1I,J, the ghrelin levels were greatly diminished upon stimulation with TNF-α. These results indicate that ghrelin is found in skin cells such as epidermal cells and macrophages, while inflammatory cytokines like TNF-α lead to loss of ghrelin expression in the skin.

### Ghrelin expression could be detected in dermis and epidermis while got diminished in both OXA-induced contact dermatitis and IMQ-induced psoriasis mouse models, and is also induced upon systemic application of ghrelin

**Figure 1:**
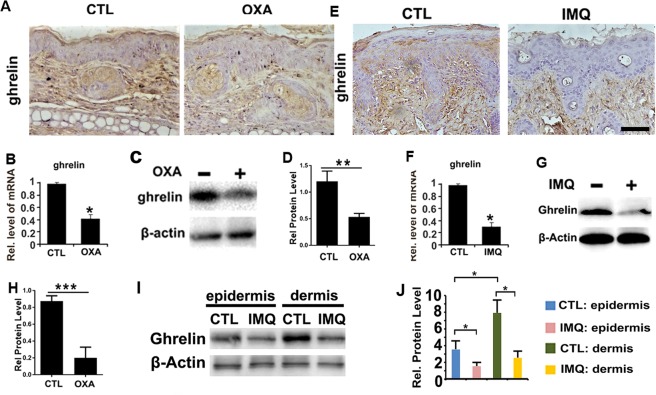
Ghrelin expression is downregulated in OXA-induced contact dermatitis and IMQ-induced psoriasis mouse models, in both epidermis and dermis. (**A**) Ghrelin expression was evaluated by immunohistochemistry, and the results show that ghrelin levels are decreased in ear tissue after stimulation by OXA. (**B**) Ghrelin levels were diminished in OXA-induced contact dermatitis models, as shown by real-time PCR. (**C**,**D**) Total protein was extracted from skin tissue, and ghrelin was reduced upon stimulation with OXA, as shown by western blotting. (**E**) Expression of ghrelin was decreased in an IMQ-induced psoriasis mouse model, as assessed by immunohistochemistry. (**F**) Ghrelin expression was greatly decreased in mice stimulated with IMQ, as shown by real-time PCR. (**G**,**H**) Expression of ghrelin is downregulated at the protein level, as shown by western blotting. (**I**) Expression level of ghrelin in epidermis and dermis of dorsal skin in CTL and IMQ groups, as was detected by western blot. (**J**) Statistical analysis for relative protein level of ghrelin based on Western blot. (*p < 0.01 vs control group). (*p < 0.05, **p < 0.01, ***p < 0.005 vs the control group). Scale bar: 150 μm.

It has been reported that ghrelin can affect many inflammatory diseases^31,32,46^, but it remains unknown whether ghrelin expression is associated with the development of contact dermatitis and psoriasis. In this study, we established mouse models of contact dermatitis and psoriasis using OXA and IMQ, respectively. Immunohistochemistry was subsequently utilized to evaluate the expression of ghrelin in OXA-induced contact dermatitis and IMQ-induced psoriasis mouse models. As shown in Fig. 1A, ghrelin secretion was decreased in contact dermatitis. Assessment of ghrelin mRNA through real-time PCR also displayed reduced ghrelin expression in the contact dermatitis model (Fig. 1B). Additionally, total protein was extracted from skin tissue of CTL and OXA + PBS experimental groups, and western blotting was performed. As shown in Fig. 1C,D, significant downregulation of ghrelin was detected in this model. In the IMQ-induced psoriasis model, immunohistochemistry revealed significant decreases in ghrelin levels compared to those in the CTL group, as shown in Fig. 1E. Similarly, real-time PCR results revealed reduced mRNA levels of ghrelin in response to stimulation by IMQ (Fig. 1F). Moreover, western blotting was performed using total protein extracted from the skin tissue of each group in experiment 2, and results demonstrated a substantial decrease in ghrelin expression in IMQ-treated mouse models (Fig. 1G,H). Following, the detailed expression pattern of ghrelin in skin was modified with seprarted dermis and epidermis tissues. As was shown in Fig. 1I,J and Supplementary Fig. 4, expression of ghrelin can be clearly detected in both dermis and epidermis. Moreover, expression level of ghrelin in epidermis is lower compared with dermis tissue, which implied that ghrelin is mainly expressed in dermis tissue. Furthermore, expression of ghrelin in both epidermis and dermis of CTL group were stronger than that of IMQ group. Additionally, systemic application of ghrelin was performed by peritoneal injection in the OXA-induced contact dermatitis mouse model followed by total protein extraction from skin tissues and western blot analysis. As shown in Supplementary Fig. 3A,B, OXA stimulation greatly decreased ghrelin levels, while systemic application of ghrelin increased its expression in dorsal skin. Moreover, mRNA was collected from each experimental group, and real-time PCR was performed. The result showed that systemic application of ghrelin clearly increased the secretion of ghrelin in skin tissue, which was initially reduced by stimulation with OXA (Supplementary Fig. 3C). Additionally, immunohistochemistry was performed in both the OXA + PBS and OXA + ghrelin groups to assess ghrelin expression. As shown in Supplementary Fig. 3D, ghrelin expression was enhanced upon systemic application of ghrelin. The decreased levels of ghrelin in skin tissue in response to both contact dermatitis and psoriasis, as well as elevation of ghrelin expression in response to systemic application of ghrelin, suggest that ghrelin might be involved in contact dermatitis and psoriasis.

### Ghrelin protects against OXA-induced contact dermatitis in a mouse model

**Figure 2:**
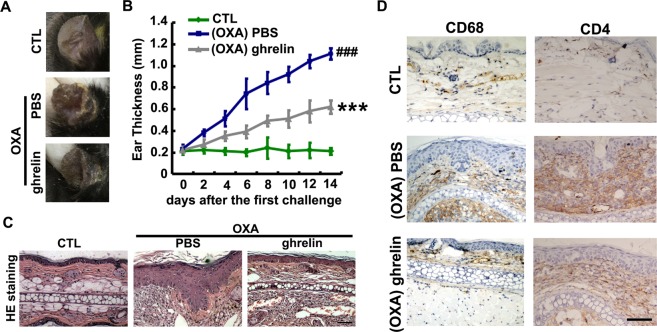
Ghrelin affects the OXA-induced mouse model of contact dermatitis morphologically and histologically. (**A**,**B**) Treatment with ghrelin significantly improves the typical signs of contact dermatitis and dramatically reduces ear thickness. (**C**) Epithelium thickness was improved in response to treatment with ghrelin in an OXA-induced contact dermatitis mouse model, as shown by HE staining. (**D**) Treatment with ghrelin led to downregulation of CD68 and CD4 expression, as shown by immunohistochemistry. (^###^p < 0.05, ***p < 0.01 vs the control group).

To identify whether ghrelin exerts a curative effect in contact dermatitis and psoriasis, an OXA-induced contact dermatitis model was established using C57BL/6 WT mice that were subsequently treated with either PBS or ghrelin. Hyperproliferation of keratinocytes and enlargement and growth of dermal capillary vasculature are the main characteristics of contact dermatitis, which illustrate both its progress and severity. First, the exterior ear lobes of each group were observed, and ear thickness was measured every 2 days beginning from the initial challenge. As shown in Fig. 2A,B, OXA stimulation resulted in faster keratinocyte hyperproliferation, as demonstrated by the appearance and thickness measurements, while treatment with ghrelin greatly improved these typical signs of contact dermatitis and dramatically reduced ear thickness. To further explore cuticle change in each experimental group, hematoxylin-eosin (HE) staining was conducted, and the results showed an attenuation of the epithelium thickness (Fig. 2C). CD68 and CD4 detection show the presence of T lymphocytes and macrophages, respectively. It is well known that both T lymphocytes and macrophages play critical roles in skin inflammatory diseases, including contact dermatitis and psoriasis. As shown in Fig. 2D, expression of CD68 and CD4 was greatly enhanced in response to stimulation by OXA; however, treatment with ghrelin drastically attenuated the secretion of CD68 and CD4, implying a reduced severity of contact dermatitis in mice. Taken together, these results indicate that ghrelin may affect the progression of contact dermatitis.

### Ghrelin inhibits the secretion of proinflammatory cytokines in OXA-induced contact dermatitis

**Figure 3:**
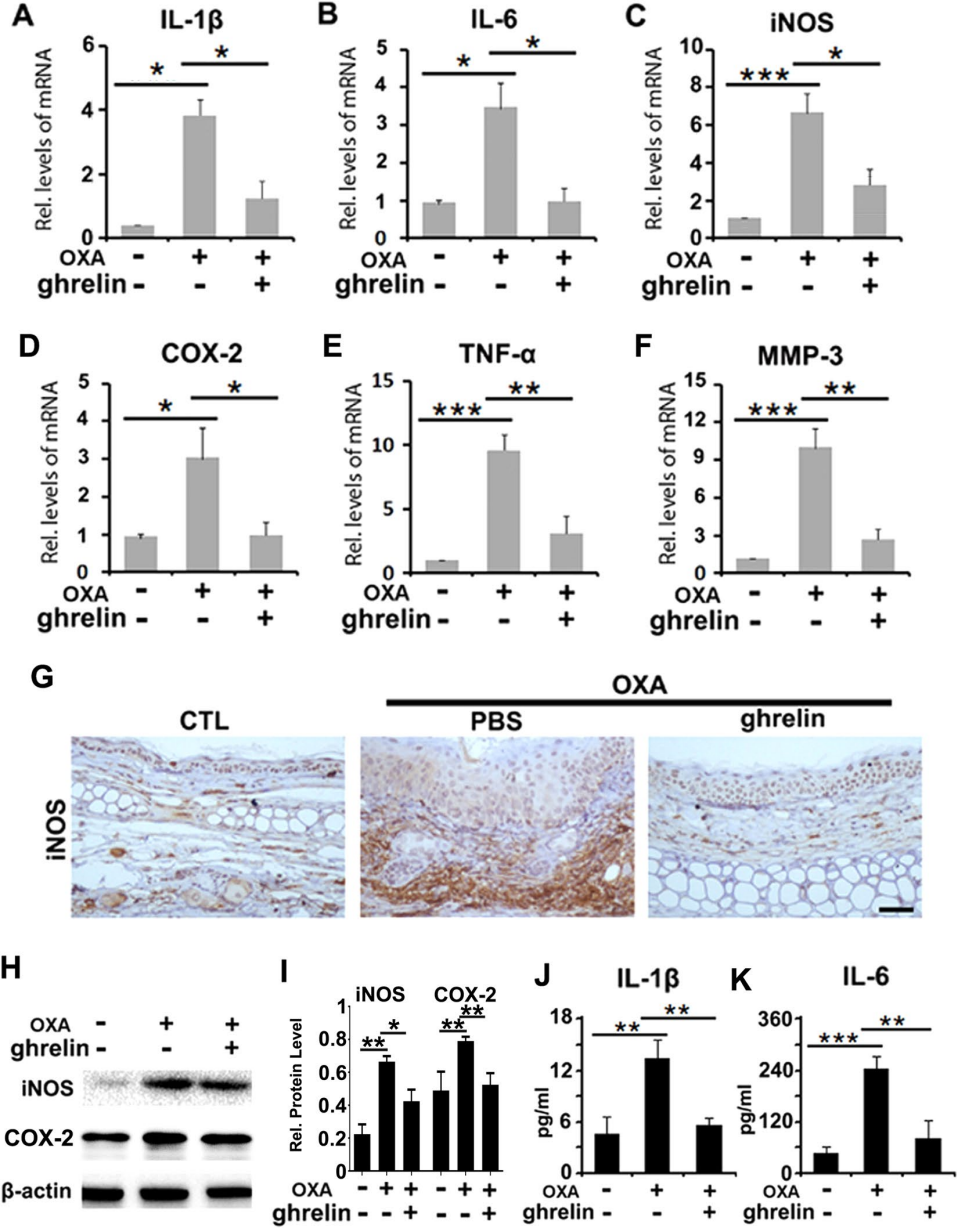
Ghrelin attenuates the secretion of proinflammatory cytokines in an OXA-induced contact dermatitis mouse model. (**A**–**F**) The levels of IL-1β, IL-6, iNOS, COX-2, TNF-α and MMP-3 were dramatically decreased upon treatment with ghrelin, as shown by real-time PCR. (**G**) iNOS expression was downregulated in mice treated with ghrelin in OXA-induced contact dermatitis, as shown by immunohistochemistry. (**H**,**I**) The protein levels of iNOS and COX-2 were markedly inhibited with ghrelin treatment, as shown by western blotting. (**J**,**K**) IL-1β and IL-6 were detected in mouse serum, indicating the ghrelin-mediated reduction in inflammatory cytokines in serum, as shown by ELISA. (*p < 0.05, **p < 0.01, ***p < 0.005 vs the control group). Scale bar: 100 μm.

Inflammatory cytokines, such as IL-1β, IL-6, iNOS, COX-2, TNF-α and MMP-3, are biomarkers that indicate inflammation severity. Assessing the expression of such cytokines in the skin lesions and serum samples of mice is essential to examine the inflammatory status of contact dermatitis. First, real-time PCR was conducted to examine the mRNA expression of IL-1β, IL-6, iNOS, COX-2, TNF-α and MMP-3, all of which were significantly increased in response to treatment with OXA, while treatment with ghrelin dramatically decreased the mRNA expression of these cytokines, as shown in Fig. 3A,F. Moreover, immunohistochemistry was used to visualize iNOS staining patterns. Lower levels of iNOS expression were observed in response to ghrelin treatment compared to that in the OXA + PBS (Fig. 3G). Additionally, western blot analysis was performed to examine the protein levels of iNOS and COX-2 for each experimental group. The results indicated that application of OXA dramatically upregulated the expression of both iNOS and COX-2; however, levels of these cytokines were downregulated in response to the application of ghrelin (Fig. 3H,I). Furthermore, as shown in Fig. 3J,K, the serum levels of IL-1β and IL-6 were greatly increased in response to treatment with OXA, an effect that was notably attenuated by ghrelin treatment.

### Ghrelin alleviates IMQ-induced psoriasiform dermatitis in mice

**Figure 4:**
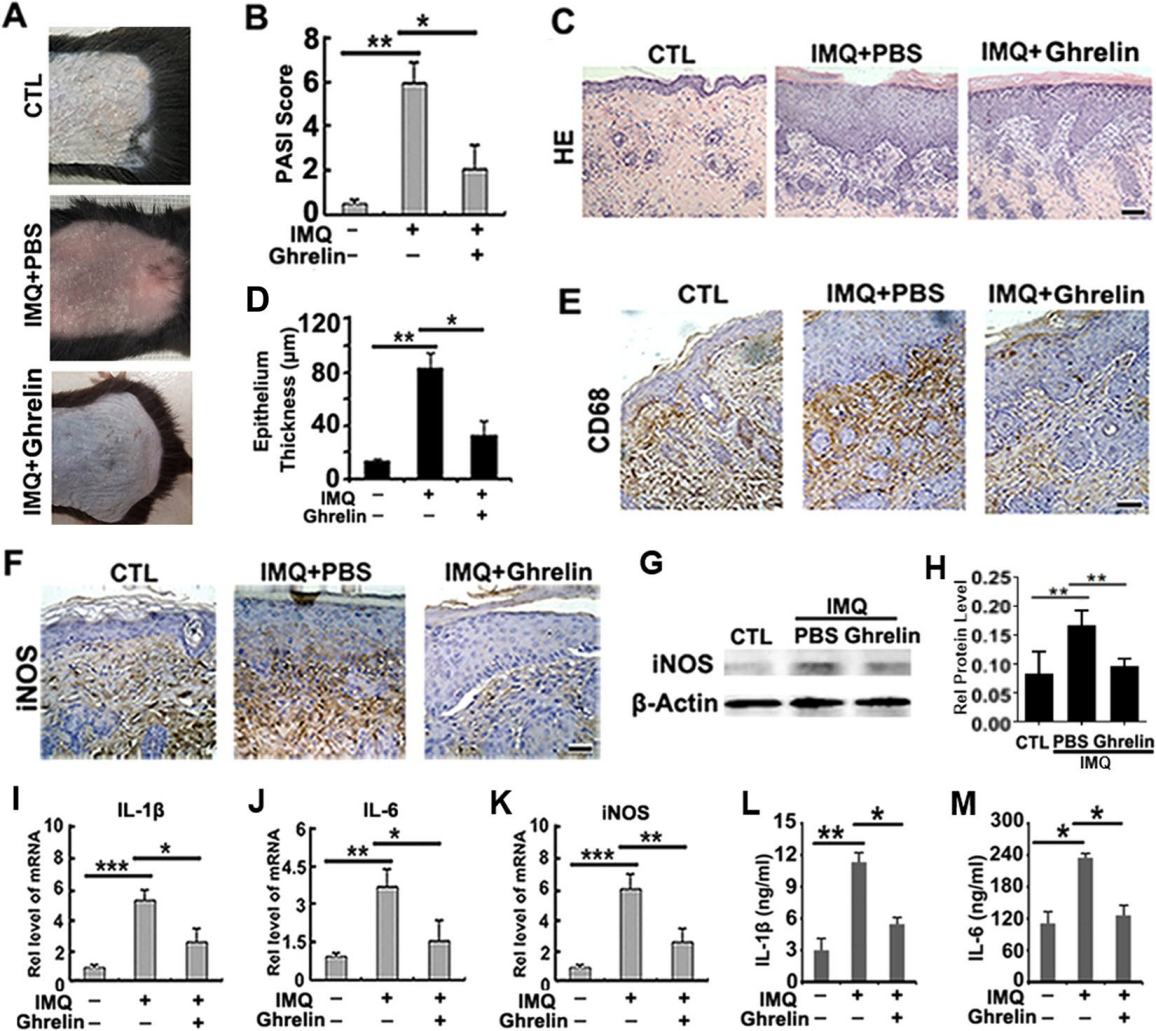
Ghrelin improves psoriasis in an IMQ-induced psoriasis mouse model. (**A**) Improved skin conditions were observed in mice treated with ghrelin. (**B**) Ghrelin treatment led to reduced PASI scores in an IMQ-treated psoriasis mouse model. (**C**,**D**) A smoother epidermis, lesser degree of parakeratosis, and reduced epidermal thickening were demonstrated upon treatment with ghrelin, as shown by both HE staining and the measurement of epithelial thickness. (**E**) Ghrelin downregulates the expression of CD68, supporting the anti-inflammatory effect of ghrelin in IMQ-induced psoriasis, as shown by immunohistochemistry. (**F**) iNOS is decreased in response to treatment with ghrelin, as shown by immunohistochemistry. (**G**,**H**) Total protein was extracted from skin tissue, and iNOS expression was decreased upon treatment with ghrelin, as shown by western blotting. (**I**–**K**) Ghrelin application significantly attenuated the mRNA expression of IL-1β, IL-6 and iNOS, as shown by real-time PCR. (**L**,**M**) The serum levels of IL-1β and IL-6 were notably reduced by ghrelin administration, as shown by ELISA. (*p < 0.01, **p < 0.01, ***p < 0.005 vs the control group). Scale bar: 150 μm.

Compared to the skin of the CTL group, the dorsal skin of mice treated with IMQ exhibited features of erythema, scaling, and thickening on the 2nd day. The lesions steadily thickened as IMQ treatment continued. Furthermore, mice treated with ghrelin exhibited reduced erythema, thinner scales, normal skin, and decreased skin thickness, and the total skin lesions were greatly reduced (Fig. 4A). The average psoriasis area and severity index (PASI) score of each group was measured and analyzed, as mentioned above. As shown in Fig. 4B, mice treated daily with IMQ exhibited greatly increased PASI scores, while IMQ mice treated with ghrelin exhibited lower PASI scores than the PBS group. Furthermore, as seen in the HE staining results, the IMQ + PBS group exhibited increased epidermal acanthosis, parakeratosis, and a higher degree of epidermal thickening, as shown in Fig. 4C. However, ghrelin significantly improved the appearance of these histological phenotypes and resulted in a smoother epidermis, reduced parakeratosis, and a lesser degree of epidermal thickening. Moreover, epithelium thickness scores of each group were measured and analyzed, as mentioned above. As shown in Fig. 4D, the mice treated daily with Vaseline (in the CTL group) exhibited no apparent changes in epithelium thickness. In contrast, the mice in the IMQ + ghrelin group exhibited reduced epithelium thickness compared with that in the IMQ + PBS group. T lymphocytes and macrophages are inflammatory cells involved in psoriasis tissue and can be detected via CD68. Studies have proved that greater expression of these two cell types indicates more-severe psoriasis^47^. To examine its expression, immunohistochemistry was performed As shown in Fig. 4E, application of IMQ greatly increased the expression of CD68, while treatment with ghrelin dramatically decreased its secretion, supporting the anti-inflammatory effect of ghrelin in IMQ-induced psoriasis.

### Ghrelin attenuates IMQ-induced changes in proinflammatory cytokines

Inflammatory cytokine expression in the skin lesions and serum samples of mice was evaluated to examine the effects of ghrelin on IMQ-induced inflammation, both locally and systemically. Immunohistochemistry was used for examination of iNOS, and the results revealed lower levels of iNOS expression in response to ghrelin treatment (Fig. 4F). Additionally, western blot analysis was conducted to examine the protein levels of iNOS in each experimental group. The result indicated that application of IMQ dramatically enhanced the iNOS expression; however, iNOS levels were downregulated upon treatment with ghrelin (Fig. 4G,H). Moreover, the mRNA expression of IL-1β, IL-6 and iNOS was greatly increased upon stimulation with IMQ, while treatment with ghrelin significantly downregulated IMQ-induced increases in the mRNA levels of these cytokines, as shown in Fig. 4I,K. In addition, as shown in Fig. 4L,M, the serum levels of IL-1β and IL-6 were greatly increased in response to treatment with IMQ but notably decreased by ghrelin administration.

### Ghrelin decreases TNF-α-mediated inflammatory reactions in RAW264.7 cells

**Figure 5:**
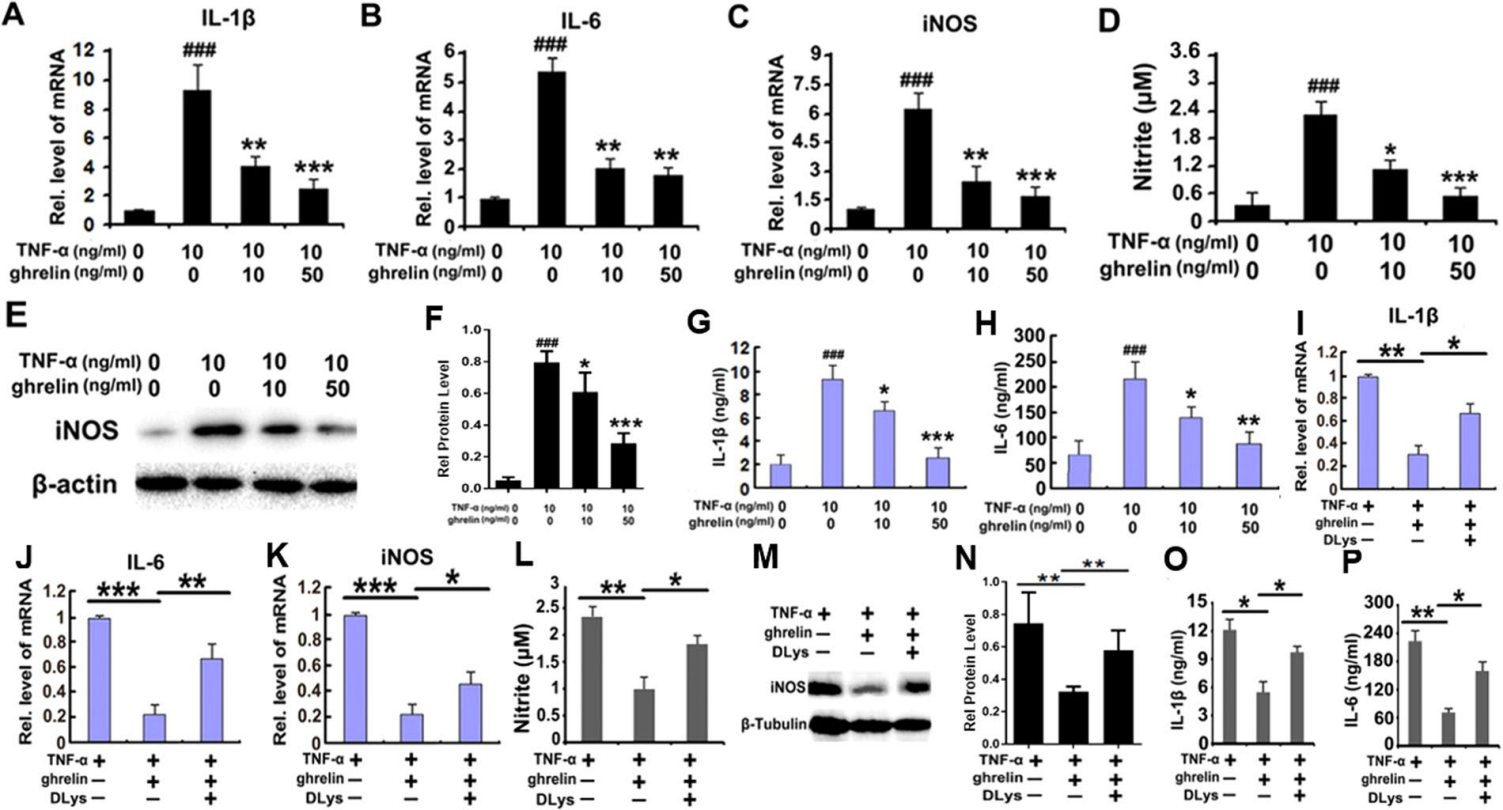
Ghrelin inhibits inflammatory reactions by combining with GHSR1a and antagonizing TNF-α signaling. (**A**–**C**) Ghrelin greatly inhibited the secretion of inflammatory cytokines, such as IL-1β, IL-6, and iNOS, *in vitro* in a dose-dependent manner, as shown by real-time PCR. (**D**) Reduced nitrite levels were promoted by the application of ghrelin in a dose-dependent manner, as shown by the Griess experiment. (**E**,**F**) iNOS expression is downregulated in response to ghrelin application in a dose-dependent manner, as shown by western blotting. (**G**,**H**) IL-1β and IL-6 secretion were changed by ghrelin pretreatment and appeared to be heightened in proportion to elevations in ghrelin concentration, as shown by ELISA. (**I**–**K**) The mRNA expression of IL-1β, IL-6 and iNOS was enhanced by application of DLys, implying that DLys antagonizes the effect of ghrelin, as shown by real-time PCR. (**L**) Addition of DLys attenuates ghrelin’s effect of decreasing nitrite production, as detected by the Griess test. (**M**,**N**) Higher iNOS protein levels occurred with DLys application, as shown by western blotting. (**O**,**P**) Application of DLys inhibited ghrelin’s antagonizing effect on the secretion of inflammatory cytokines IL-1β and IL-6 induced by stimulation with TNF-α, as shown by ELISA. (*p < 0.005, **p < 0.05, ***p < 0.005, ^###^p < 0.01 vs the control group). Scale bar: 100 μm.

Further experiments were conducted in this study to examine whether ghrelin inhibits the inflammatory reactions initiated by TNF-α *in vitro*. Western blotting and PCR were performed to examine the protein levels and mRNA expression of inflammatory cytokines, respectively, and the Griess experiment was performed to examine nitrite levels after stimulation with TNF-α. The results showed that the mRNA levels of inflammatory cytokines, including IL-1β, IL-6, and iNOS, were markedly induced in RAW264.7 cells after stimulation with TNF-α but significantly decreased in response to incubation with ghrelin in a dose-dependent manner (Fig. 5A,C). The Griess experiment was conducted, and the nitrite stimulation by TNF-α was suppressed in response to ghrelin treatment in a dose-dependent manner (Fig. 5D). Additionally, iNOS expression was evaluated through western blotting. As illustrated in Fig. 5E,F, TNF-α stimulation resulted in higher iNOS levels compared to those in the other groups, indicating that TNF-α treatment induced the secretion of inflammatory cytokines, such as iNOS, while treatment with ghrelin attenuated these effects in a dose-dependent manner. Moreover, the cell culture supernatant was collected after a 1-day incubation to examine the concentrations of IL-1β and IL-6 through enzyme-linked immunosorbent assays (ELISAs). IL-1β and IL-6 secretion was enhanced by stimulation with TNF-α but was decreased by ghrelin treatment, with heightened effects in response to elevated ghrelin concentrations (Fig. 5G,H).

To identify GHSR1a’s effect on ghrelin’s TNF-α-mediated anti-inflammatory properties, DLys, a well-accepted GHSR1a blocker, was applied to RAW264.7 cells along with stimulation of TNF-α and treatment with ghrelin. Subsequently, mRNA expression of IL-1β, IL-6 and iNOS was significantly upregulated in response to TNF-α stimulation, while being significantly downregulated by ghrelin. Moreover, application of DLys seemed to antagonize the effect of ghrelin (Fig. 5I,K). The Griess experiment showed that the addition of DLys reduced ghrelin’s effect on the production of nitrite, as shown in Fig. 5L. Additionally, the western blot results showed a higher grayscale level in the ghrelin treatment group without DLys than in the ghrelin + DLys application group, indicating a greater extent of inflammation (Fig. 5M,N). ELISA was also used on the culture medium collected from each experimental group to examine the expression of IL-1β and IL-6. As shown in Fig. 5O,P, ghrelin attenuated the levels of inflammatory cytokines induced by stimulation with TNF-α, while application of DLys reduced these effects. These results indicate that ghrelin plays a curative role in inflammation mainly by combining with GHSR1a and that GHSR1a plays a critical role with ghrelin in exerting its anti-inflammatory effects through antagonizing the TNF-α signaling pathway.

### Effects of ghrelin on NF-κB signaling in contact dermatitis and psoriasis

**Figure 6:**
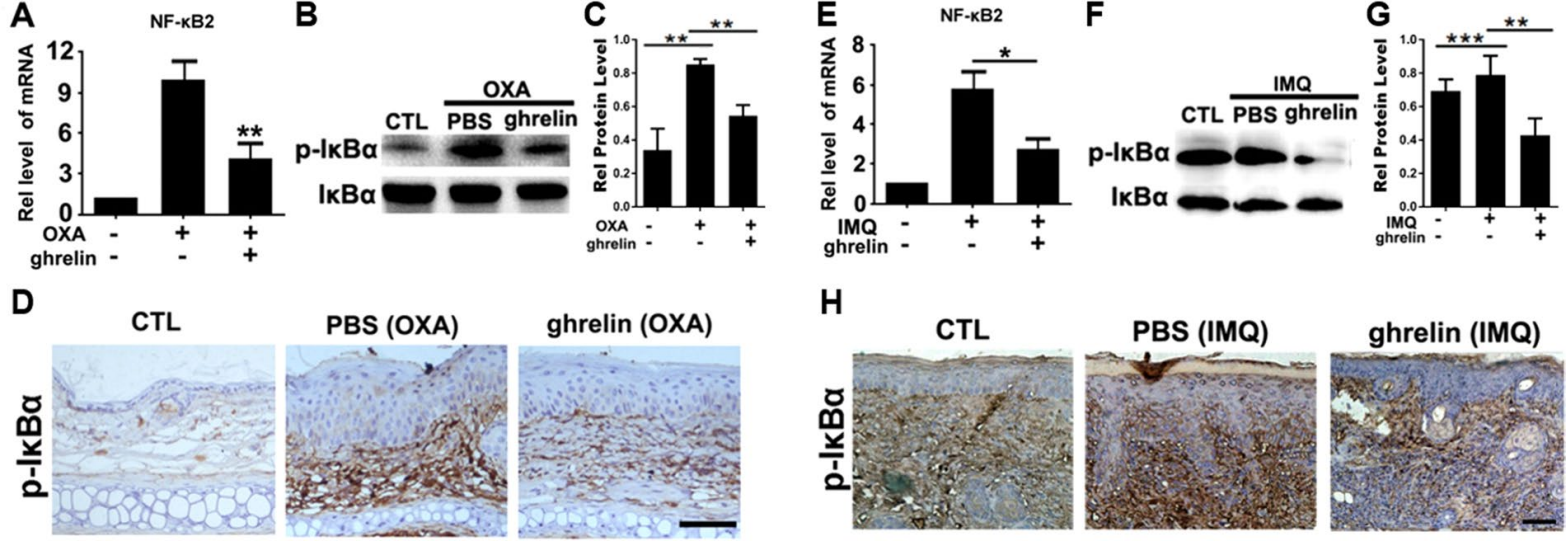
Ghrelin exerts its anti-inflammatory effects on contact dermatitis and psoriasis through NF-κB signaling. (**A**) Expression of mRNA NF-κB2 is suppressed upon treatment with ghrelin in an OXA-treated contact dermatitis mouse model. (**B**,**C**) The significantly increased p-IκBα in response to stimulation by OXA was markedly inhibited by ghrelin, as shown by western blotting. (**D**) Expression of p-IκBα in ear samples was significantly enhanced by OXA stimulation, while ghrelin decreased p-IκBα levels, as shown by immunohistochemistry. (**E**) mRNA levels of NF-κB2 are markedly decreased by ghrelin treatment in an IMQ-induced psoriasis mouse model, as shown by real-time PCR. (**F**,**G**) Significantly decreased expression of p-IκBα in the total protein extracted from skin tissue was detected after application of ghrelin in the IMQ-induced psoriasis mouse model, as detected by western blotting. (**H**) p-IκBα is significantly decreased upon treatment with ghrelin in the psoriasis mouse model, as shown by immunohistochemistry. (*p < 0.05, **p < 0.01, ***p < 0.005 vs the control group). Scale bar: 100 μm.

The effects of ghrelin on the downstream signaling factors induced by TNF-α in the OXA and IMQ mouse models were further examined. In the OXA-induced contact dermatitis mouse model, after collecting samples from each experimental group, mRNA was extracted, and levels of NF-κB2 were examined. As shown in Fig. 6A, the expression of NF-κB2 mRNA was suppressed in response to ghrelin treatment, indicating its potential ability to suppress the NF-κB signaling pathway. Moreover, western blot analysis of p-IκBα and IκBα proteins was performed. The results in Fig. 6B,C show significantly increased expression of p-IκBα in the mice stimulated with OXA, and this change was markedly inhibited by ghrelin. The expression of p-IκBα in ear samples was clearly enhanced by OXA stimulation but decreased in response to ghrelin, as illustrated in Fig. 6D. Additionally, in the IMQ-treated psoriasis mouse model, as shown in Fig. 6E, the mRNA levels of NF-κB2 were greatly reduced upon treatment with ghrelin, as shown by real-time PCR. Furthermore, the expression of p-IκBα protein extracted from skin tissue was also greatly decreased upon treatment with ghrelin, indicating reduced activation of NF-κB signaling in response to ghrelin treatment (Fig. 6F,G). Moreover, p-IκBα expression was also detected in each experimental group through immunohistochemistry, and significantly decreased p-IκBα expression was detected after application of ghrelin, further showing ghrelin’s antagonistic effect on NF-κB (Fig. 6H). In summary, downregulation of p-IκBα levels upon treatment with ghrelin in OXA-induced contact dermatitis and IMQ-induced psoriasis mouse models indicate ghrelin’s ability to antagonize NF-κB signaling in these disorders.

### A possible mechanism of ghrelin in contact dermatitis and psoriasis involves antagonizing the TNF-α and NF-κB signaling pathways

**Figure 7:**
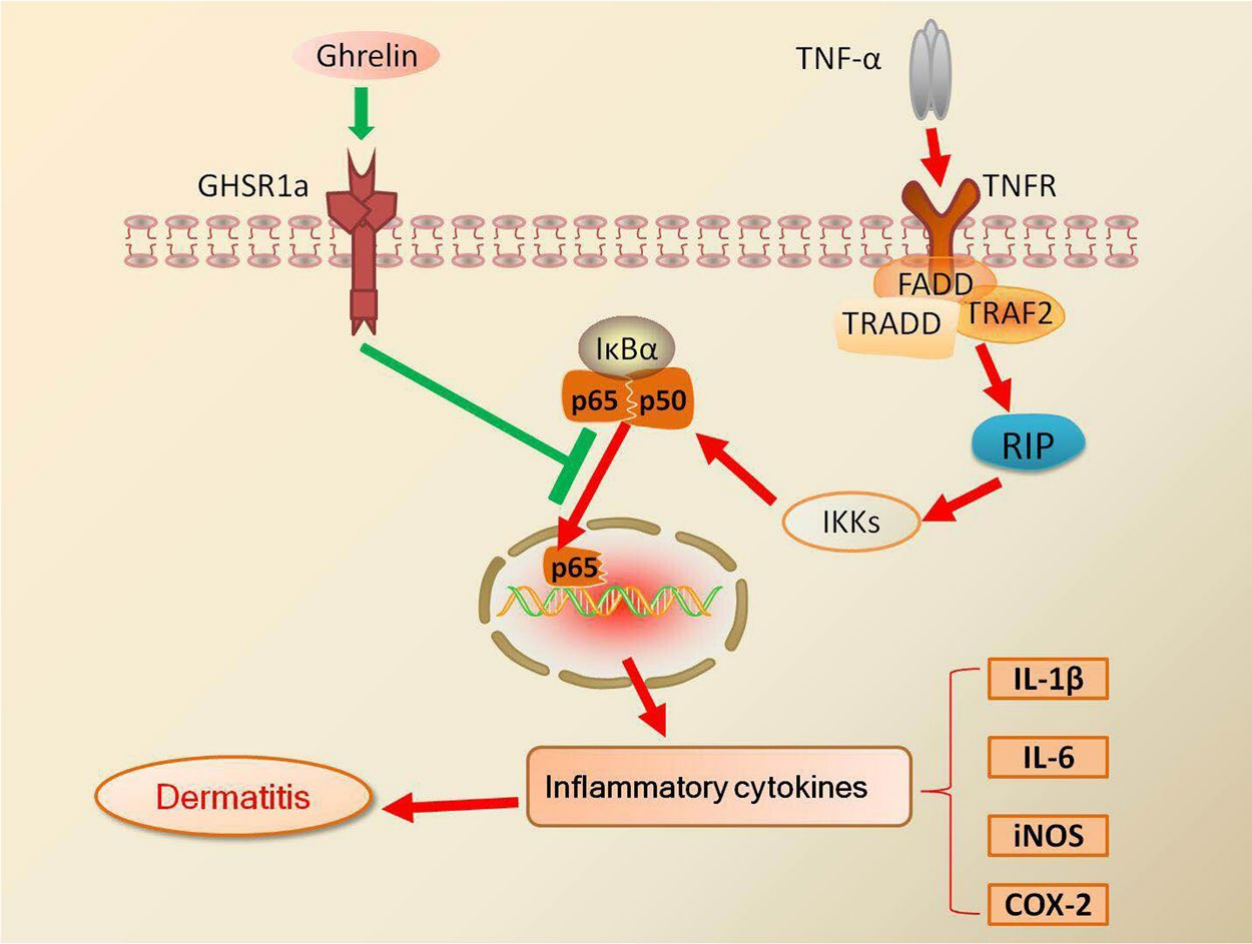
Schematic depicting ghrelin’s proposed effect on contact dermatitis and psoriasis. TNF-α binds to TNFR, which activates IKK to phosphorylate and degrade IκBα. This change results in activation of the NF-κB signaling pathway, causing the secretion of proinflammatory cytokines and subsequent inflammation. Ghrelin exerts its effects through binding to GHSR1a, followed by antagonizing TNF-α, resulting in suppression of NF-κB signaling. This signaling pathway represents a potential therapeutic method for both contact dermatitis and psoriasis.

TNF-α binds to TNFR, followed by subsequent activation of IKK. Activated IKK then phosphorylates and degrades IκBα, which can activate NF-κB signaling to mediate inflammatory reactions^48,49^. In this study, we confirmed that ghrelin exerts its effects through binding to GHSR1a, subsequently antagonizing TNF-α signaling and attenuating activation of the NF-κB signaling pathway. Suppression of NF-κB activation decreases the secretion of inflammatory cytokines, resulting in curative effects on dermatitis, including contact dermatitis and psoriasis, as shown in Fig. 7.

## Discussion

Ghrelin, a recently discovered novel peptide, has been reported to have dramatic effects on inflammation^29,32,35^. Previous reports have shown that ghrelin not only mediates inhibition of inflammatory cytokines but also acts as a gastrointestinal peptide to stimulate appetite^50,51^. This action makes ghrelin a potential anti-inflammatory factor that has attracted much attention and has been extensively researched. Skin inflammatory diseases, such as contact dermatitis and psoriasis, affect millions of people all over the world, resulting in reduced quality of life^52–56^. However, the mechanisms and cure for contact dermatitis and psoriasis remain unknown^4,57–59^. Many cell types, including epidermal cells^60^, lymphocytes^61^, macrophages^62^, fibroblasts^63^ and dendritic cells^64^, secrete ghrelin, and these cells have been reported to mediate effects on skin inflammation, including contact dermatitis and psoriasis^65–67^, indicating the possibility that ghrelin may affect contact dermatitis and psoriasis. However, the effectiveness and mechanism of ghrelin on these skin inflammatory diseases have not been explored. Many reports have suggested that TNF-α is critical in the pathogenesis of contact dermatitis and psoriasis; therefore, therapeutic strategies based on TNF-α may be useful for these diseases^23,68^. Herein, we examined expression of ghrelin in both dermis and epidermis, as well as in a number of skin cell types including macrophage, skin fibroblast and epidermal cells. Additionally, we found decreased levels of ghrelin in the skin in contact dermatitis and psoriasis, and these modifications are detected in both dermis and epidermis tissues, which are primarily caused by the inflammatory cytokine secretion by macrophages in skin tissue. The results also showed that the inflammatory condition of macrophage, skin fibroblast and epidermal cells may also lead to decreased expression of ghrelin in skin. Moreover, this study implies that ghrelin alleviates OXA-induced contact dermatitis, IMQ-induced psoriasiform dermatitis, and TNF-α-induced inflammation in RAW264.7 cells by antagonizing TNF-α signaling, indicating that ghrelin may facilitates treatments for contact dermatitis and psoriasis through TNF-α.

Contact dermatitis is a skin disease linked to allergy^69^. However, it has also been proved to be associated with inflammatory factors^70^. Moreover, treatment aimed at reducing inflammation has been reported to be clinically effective against contact dermatitis^71^. The OXA-induced contact dermatitis mouse model in mouse ear lobes is widely accepted and applied as a model of human contact dermatitis and was used in this study to examine the inhibitory effect of ghrelin in contact dermatitis^29,72^. Experiments in this study illustrate that treatment with ghrelin greatly decreased OXA-induced contact dermatitis, as indicated by the comparison of the morphological and histopathological features between the groups. Decreased levels of CD68 and CD4 in response to treatment with ghrelin caused decreased levels of T cells and macrophages, implying a reduced hypersensitivity reaction^73–75^. Inflammatory cytokines, including IL-1β, IL-6, iNOS, COX-2, TNF-α and MMP-3, are critical mediators in the inflammatory response in contact dermatitis^76–78^. In line with preceding studies, the results in this study identified that the expression of these inflammatory cytokines was greatly increased in the skin lesions and sera of OXA-induced contact dermatitis mouse models. Furthermore, treatment with ghrelin decreased the levels of these cytokines, illustrating that ghrelin exerts protective effects on contact dermatitis by alleviating inflammatory reactions.

As a well-accepted mouse model of psoriasis, IMQ administration can lead to psoriasis-like symptoms, including erythema, scaling, and skin thickening, and this model has been proven to be a credible platform for researching psoriasis treatments^79^. In this study, we demonstrate that ghrelin diminishes these symptoms, signifying that ghrelin may be effective against psoriasis. Additionally, for this skin disease mainly caused by inflammatory reactions, inflammatory cytokines, including IL-1β, IL-6 and iNOS, are critical mediators and are increased in psoriasis^58,80^. Herein, ghrelin treatment led to greatly reduced levels of these molecules in an IMQ-induced psoriasis mouse model, which corresponded to a reduced severity of psoriasis. Taken together, these studies corroborate ghrelin’s possible effect as a curative agent for psoriasis.

Macrophages are present during and play a critical role in the development of skin inflammation in both contact dermatitis and psoriasis. Additionally, macrophages are one of the main targets in the treatment of these two diseases^81–83^. In this study, we demonstrated that macrophages are one of the main cell types secreting ghrelin in skin tissue and demonstrated that the inflammatory cytokines expressed by macrophages greatly diminished the secretion of ghrelin in the skin, which may lead to exacerbated contact dermatitis and psoriasis. TNF-α has been studied as one of the main inducers of skin diseases, including contact dermatitis and psoriasis. As previously reported, TNF-α acts as a mediator in the process of inflammation^12,84,85^. Studies have shown increased levels of TNF-α in the skin tissues and blood samples of contact dermatitis and psoriasis patients^86,87^, and treatments that inhibit activation of TNF-α display significant efficacy in both contact dermatitis and psoriasis^88–91^. Stimulation of TNF-α enhances the secretion of a number of inflammatory cytokines, including IL-1β, IL-6 and iNOS, which are essential inflammatory biomarkers for examining the severity of inflammation^92–94^. In this study, RAW264.7 cells were stimulated with TNF-α, and the inflammatory cytokines mentioned above were elevated upon stimulation but diminished in response to treatment with ghrelin. Decreased levels of TNF-α-associated inflammatory biomarkers highlight ghrelin’s inhibition of the inflammation induced by TNF-α. Additionally, application of the GHSR1a blocker DLys greatly attenuated the effect of ghrelin treatment, signifying that ghrelin affects TNF-α signaling by combining with GHSR1a. These studies imply that ghrelin exerts anti-inflammatory effects *in vitro* by interacting with GHSR1a and antagonizing TNF-α function.

NF-κB signaling is a key pathway in TNF-α-induced inflammatory reactions^49,85,95^. TNF-α activate IKK, leading to the phosphorylation and degradation of IκB and the subsequent activation of the NF-κB signaling pathway, which causes inflammatory reactions^96,97^. Furthermore, TNF-α plays a critical role in skin inflammation in contact dermatitis and psoriasis^98–100^. In this study, the effect of ghrelin on NF-κB signaling was examined in OXA-induced contact dermatitis and IMQ-induced psoriasis mouse models, as well as TNF-α-treated RAW264.7 cells. Results demonstrated that administration of ghrelin greatly repressed NF-κB signaling activity. These findings suggest that ghrelin may perform its protective role in both contact dermatitis and psoriasis by barring activation of the NF-κB signaling pathway.

In summary, these findings suggest that ghrelin might be protective in contact dermatitis and psoriasis. Additionally, treatment with ghrelin contributes to marked decreases in the inflammation severity induced by TNF-α, which are mediated through ghrelin binding to GHSR1a. Furthermore, ghrelin seems to exert anti-inflammatory effects in contact dermatitis and psoriasis through suppression of NF-κB signaling, which acts as downstream of TNF-α. This study suggests that ghrelin may be a potential therapeutic candidate for both contact dermatitis and psoriasis treatment and may represent a novel treatment for skin inflammatory diseases.

## Supplementary information

Supplementary Material
