# Supplementary material for: Ghrelin protects against contact dermatitis and psoriasiform skin inflammation by antagonizing TNF-α/NF-κB signaling pathways
Source: Sci Rep. 2019 Feb 4;9:1348. doi: 10.1038/s41598-018-38174-2 (PMC6362006; doi:10.1038/s41598-018-38174-2)
Supplement: Supplementary file 1 — Supplementary Material [file 41598_2018_38174_MOESM1_ESM.pdf]

Ghrelin protects against contact dermatitis and psoriasiform skin inflammation by antagonizing TNF-α/NF-κB signaling pathways

Ruize Qu[⁋1,2], Xiaomin Chen[⁋1,2], Jing Hu[1,2], Yufeng Fu[2], Jiangfan Peng[2], Yuhua Li[4], Jingxi Chen[3], Peng Li[1], Long Liu[1], Jiankang Cao[4], Wenhan Wang[2,4], Cheng Qiu[2], Linlin Guo[2], Krasimir Vasilev[5], Jianying Chen[6], Gengyin Zhou[1], Weiwei Li[1,]* and Yunpeng Zhao[4,]*

[1] Department of Pathology, Qilu Hospital, Shandong University, Jinan, China;

[2] Cheeloo College of Medicine, Shandong University, Jinan, China;

[3] Department of Obstetrics and Gynecology, Qilu Hospital, Shandong University, Jinan, China;

[4] Department of Orthopedics, Qilu Hospital, Shandong University, Jinan, China;

[5] School of Engineering, University of South Australia, Mawson Lakes, South Australia, Australia;

[6] Institute of Biopharmaceuticals of Shandong Province, Jinan, China;

⁋These authors contributed equally to this article.

*** Correspondence:**
Correspondence to Weiwei Li:

Department of Pathology, Qilu Hospital, Shandong University, 107 Wenhuaxi Rd., Jinan 250012, China.
liweiweizhao@163.com

Correspondence to Yunpeng Zhao:

Department of Orthopedics, Qilu Hospital, Shandong University, 107 Wenhuaxi Rd., Jinan 250012, China.

miraculously2008@163.com

This file includes

Supplementary Table 1

Supplementary Figure1-4

Supplementary Tables

Table 1. Primers of Real-time PCR

Source	Primer	Forward	Reverse
Mouse	IL-1β	5’-GAAATGCCACCTTTTGACAGTG-3’	5’-TGGATGCTCTCATCAGGACAG-3’
	IL-6	5’-CTGCAAGAGACTTCCATCCAG-3’	5’-AGTGGTATAGACAGGTCTGTTGG-3’
	iNOS	5’-CTCTTCGACGACCCAGAAAAC-3’	5’-CAAGGCCATGAAGTGAGGCTT-3’
	NF-κB 2	5’-TGGCATCCCCGAATATGATGA-3’	5’-TGACAGTAGGATAGGTCTTCCG-3’
	p-IκBα	5’-ATGCAGAGTACCACTAACTACCT-3’	5’-CCTCCCCGGATTTCTTGTTTC-3’
	GAPDH	5′-AGCAGTCCCGTACACTGGCAAAC-3′	5′-TCTGTGGTGATGTAAATGTCCTCT-3′

Supplementary Figure Legend

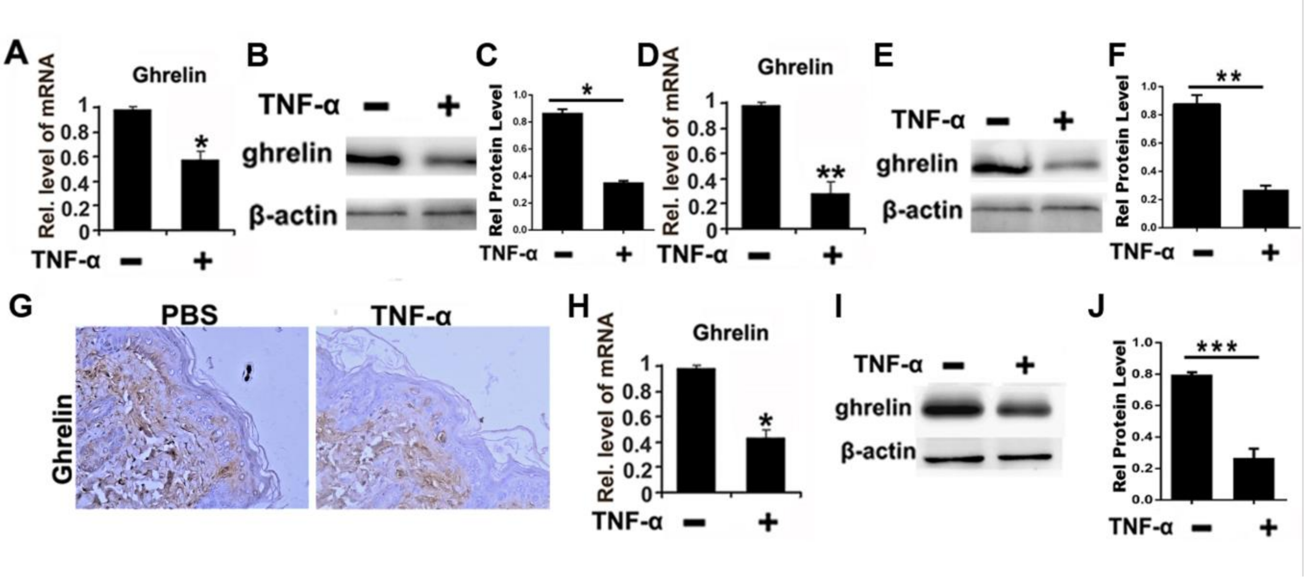

Supplementary Figure 1. Expression pattern of ghrelin in RAW264.7 cell, NHEK cell and ex vivo skin tissue cultured in stimulation of TNF-α. A) Ghrelin was detected in RAW264.7 cell, and ghrelin level was diminished with the stimulation of TNF-α, as measured by real-time PCR. **B-C)** Total protein was extracted from RAW264.7 cell of each group, and ghrelin level was diminished by TNF-α. **D)** Ghrelin was detected in NHEK cell, and TNF-α diminished production of ghrelin, as assayed by real-time PCR. **E-F)** ghrelin was expressed in NHEKs, and TNF-α downregulated expression level of ghrelin, as detected by western blot. **G)** Ghrelin was found in ex vivo culture of skin tissues, while TNF-α treatment suppressed expression level of ghrelin, as assayed by immunohistochemistry. **H)** Skin tissue was collected from each group, and ghrelin expression was found in skin tissues, while TNF-α stimulation repressed production of ghrelin in skin tissue, as measured by real-time PCR. **I-J)** Ghrelin was detected in skin tissue, which was diminished by TNF-α stimulation, as assayed by western blot. *p<0.001, **p<0.05, ***p<0.005 vs control group. Scale bar: 100μm.

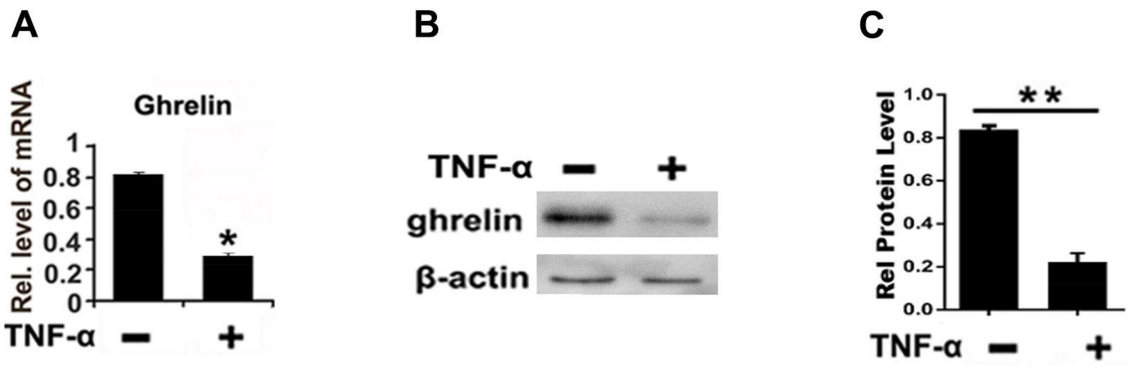

Supplementary Figure 2. Ghrelin can be detected in fibroblast cell and got diminished with the stimulation of TNF-α.A) Ghrelin was detected in fibroblast cell, which showed ghrelin level was diminished with the stimulation of TNF-α, as measured by real-time PCR. **B-C)** Total protein was gathered from fibroblast cell, and ghrelin level was diminished by TNF-α.

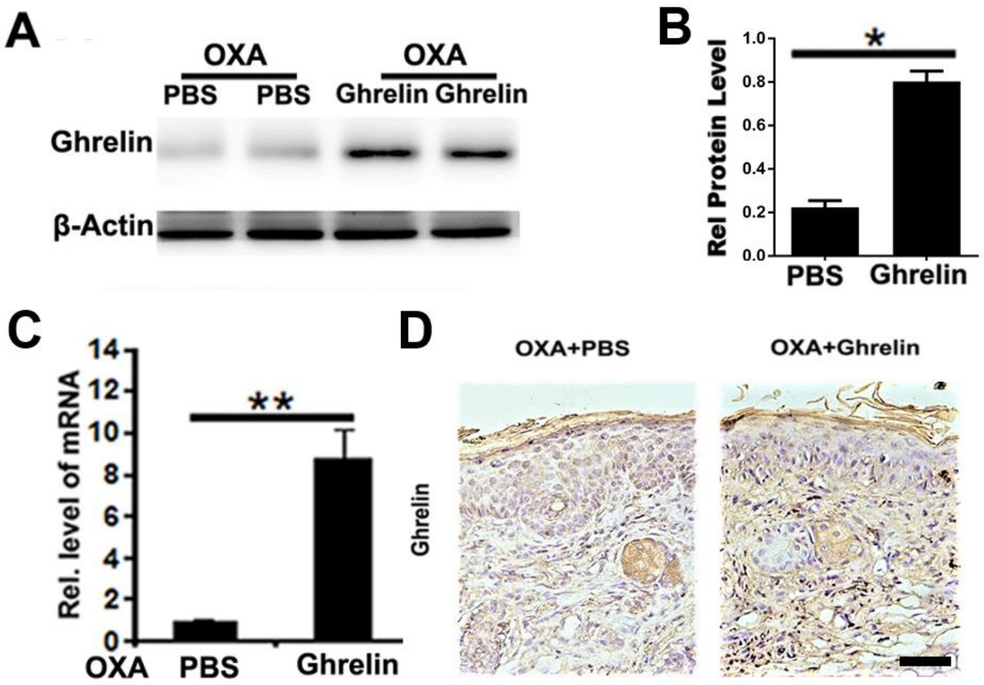

Supplementary Figure 3. Ghrelin secretion in skin got upregulated with the systemic application of ghrelin. A-B) Systemic application of ghrelin increased the expression of ghrelin in ear skin stimulated with OXA, detected by western blot. **C)** Production of ghrelin in skin tissue was enhanced by ghrelin, as assayed by real-time PCR. **D)** Stimulation of OXA diminished ghrelin level in ear skin tissues, while systemic application of ghrelin abolished the effect of OXA, as detected by immunohistochemistry. *$p<0.01$, **$p<0.005$ vs control group. Scale bar: 150μm.

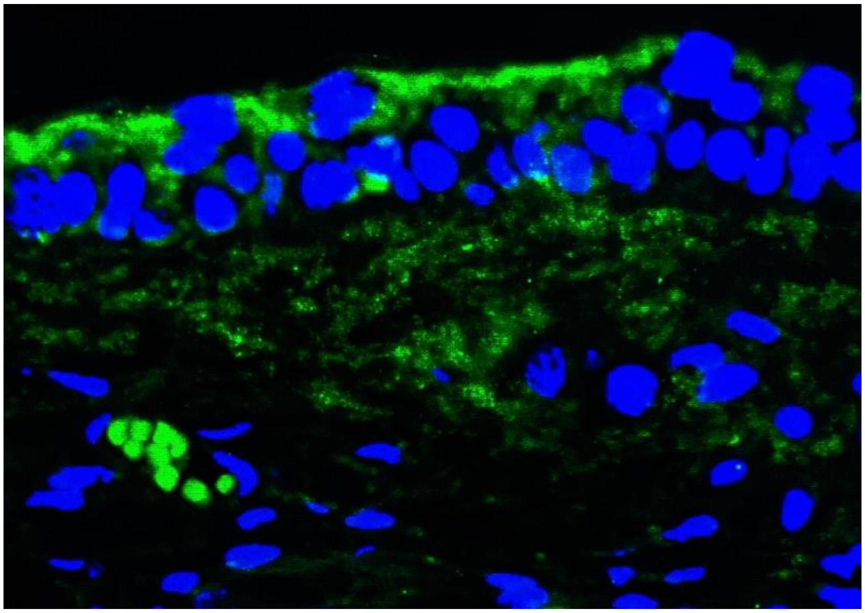

Supplementary Figure 4. Detection of ghrelin in skin tissues through immunofluorescence. Expression of ghrelin can be found in both epidermis and dermis.
